# Gloss perception: Searching for a deep neural network that behaves like humans

**DOI:** 10.1167/jov.21.12.14

**Published:** 2021-11-24

**Authors:** Konrad Eugen Prokott, Hideki Tamura, Roland W. Fleming

**Affiliations:** 1 Department of Experimental Psychology, Justus-Liebig-University Giessen, Giessen, Germany; 2 Department of Computer Science and Engineering, Toyohashi University of Technology, Toyohashi, Aichi, Japan; 3 Japan Society for Promotion of Sciences, Chiyoda, Tokyo, Japan; 4 Center for Mind, Brain and Behavior (CMBB), University of Marburg and Justus Liebig University Giessen, Germany

**Keywords:** neural networks, gloss perception, material perception

## Abstract

The visual computations underlying human gloss perception remain poorly understood, and to date there is no image-computable model that reproduces human gloss judgments independent of shape and viewing conditions. Such a model could provide a powerful platform for testing hypotheses about the detailed workings of surface perception. Here, we made use of recent developments in artificial neural networks to test how well we could recreate human responses in a high-gloss versus low-gloss discrimination task. We rendered >70,000 scenes depicting familiar objects made of either mirror-like or near-matte textured materials. We trained numerous classifiers to distinguish the two materials in our images—ranging from linear classifiers using simple pixel statistics to convolutional neural networks (CNNs) with up to 12 layers—and compared their classifications with human judgments. To determine which classifiers made the same kinds of errors as humans, we painstakingly identified a set of 60 images in which human judgments are consistently decoupled from ground truth. We then conducted a Bayesian hyperparameter search to identify which out of several thousand CNNs most resembled humans. We found that, although architecture has only a relatively weak effect, high correlations with humans are somewhat more typical in networks of shallower to intermediate depths (three to five layers). We also trained deep convolutional generative adversarial networks (DCGANs) of different depths to recreate images based on our high- and low-gloss database. Responses from human observers show that two layers in a DCGAN can recreate gloss recognizably for human observers. Together, our results indicate that human gloss classification can best be explained by computations resembling early to mid-level vision.

## Introduction

Recognizing materials from their visual appearance is an important task for the human visual system (Adelson, 2001; Anderson, 2011; Fleming, 2014; Fleming, 2017; Komatsu & Goda, 2018). One particularly interesting aspect of material perception is the perception of gloss (Chadwick & Kentridge, 2015; Marlow, Kim, & Anderson, 2012; Nishida & Shinya, 1998). From judging the freshness of food to recognizing a wet and slippery spot on the ground, gloss perception is an important daily task. Yet, despite its importance, the computations underlying human perception of gloss remain poorly understood (Anderson, 2020).

The appearance of an object results from an interaction between the shape of the object, the illumination, and the optical properties of the object. Material perception poses the visual system with the task of separating the contributions of these factors so that it can recognize the characteristic optical properties of a material among differently shaped objects and in a large range of environments. This is not a trivial task, as any given object can cause a wide range of retinal images depending on its viewing conditions, while objects made of different materials can yield very similar images.

Previous models of gloss perception range from very simple summary statistics—such as histogram skewness (Motoyoshi, Nishida, Sharan, & Adelson, 2007; Sawayama & Nishida, 2018) and contrast in particular frequency subbands (Boyadzhiev, Bala, Paris, & Adelson, 2015)—to the idea that sophisticated photogeometric computations determine the causal origin of features (e.g., in distinguishing highlights from texture markings) (Anderson & Kim, 2009; Kim, Marlow, & Anderson, 2011). Claims that statistics of the luminance distributions in an image can explain human gloss perception have been contradicted by several studies showing the importance of spatial information and the congruence of specular highlights with shading patterns (Anderson & Kim, 2009; Beck & Prazdny, 1981; Kim & Anderson, 2010; Kim et al., 2011; Marlow, Kim, & Anderson, 2011; Todd, Norman, & Mingolla, 2004). Indeed, identical image gradients can be interpreted as glossy or matte, depending on the apparent three-dimensional (3D) surface structure (Marlow & Anderson, 2015; Marlow, Todorović, & Anderson, 2015), reiterating in the field of gloss perception what has been suggested for several decades in the field of lightness perception (Gilchrist, 1977). Like lightness perception, several authors place material perception—or, more specifically, gloss perception—as a task of mid-level vision (Fleming, 2014; Kim, Marlow, & Anderson, 2012; Liu, Sharan, & Rosenholtz, 2010; Sharan, Liu, Rosenholtz, & Adelson, 2013). Mid-level vision concerns the pooling and comparison of low-level image features such as orientation, color, brightness, or scale with intermediate-level representations of surface structure, such as local surface geometry, usually with the assumption that the output of such processes disentangles physical causes that are comingled in the input. In using mid-level features, the human visual system makes use of heuristics for gloss perception and becomes susceptible to misperceptions (see also Fleming, 2012). Marlow et al. (2012) have shown that perceived gloss varies with perceived contrast, coverage, and sharpness of highlights. It has also been shown that shape and illumination can influence perceived gloss (Fleming, Dror, & Adelson, 2003; Ho, Landy, & Maloney, 2008; Olkkonen & Brainard, 2011). It is important for a good candidate model of human gloss perception to capture not just the broad successes of the visual system but also the misperceptions specific to humans.

Arguably, the most fundamental gloss perception task is to distinguish categorically whether or not a given surface is glossy. Given the enormous diversity of images that can be created by glossy and non-glossy surfaces, this is a non-trivial inference. While previous studies have generally investigated gloss perception within relatively constrained stimulus sets, here we took a “big data” approach to the gloss classification task, using machine learning techniques to train different classifiers on a large set of images. This way, we fit our models to a dataset that encompasses large variations in the appearance that high- or low-gloss materials can take, allowing our models to identify features that are diagnostic over a wide range of appearances. Using computer graphics, we rendered a large dataset of high- and low-gloss (near matte) textured materials under the same viewing conditions. We then compared model classifications to human judgments. We tested a range of different model classes to determine which ones best predict human responses. While research in machine learning typically focuses on achieving the best possible performance at a task, here our focus is on identifying which models reproduce the specific characteristics of *human* gloss judgments, spanning both their errors and successes of gloss classification.

To test how much of human gloss discrimination can be explained by simple image features we created two hand-engineered models based on summary pixel statistics and texture statistics. The first of these was a support vector machine (SVM) trained on eight simple pixel summary statistics (mean, variance, skewness, and kurtosis of pixel luminance and saturation histograms). The other was a logistic regression classifier trained on texture statistics from Portilla and Simoncelli (2000). These mid-level visual features capture color and higher order wavelet coefficient statistics, which have been used to model human perception of texture and aspects of peripheral vision (Freeman & Simoncelli, 2011).

We also look at feedforward convolutional neural networks (CNNs). CNNs have dominated computer vision benchmark tests for object recognition for nearly a decade (Kietzmann, McClure, & Kriegeskorte, 2019) and have achieved approximately human level performance (VanRullen, 2017). There have been links drawn between CNNs and the human visual system—from models that are architecturally inspired by our knowledge of the ventral stream (Kubilius et al., 2019; Simonyan & Zisserman, 2015; Spoerer, McClure, & Kriegeskorte, 2017) to analyses that compare responses of CNNs or representations within networks to human behavioral data (e.g., Cichy, Khosla, Pantazis, Torralba, & Oliva, 2016; Kriegeskorte, Mur, & Bandettini, 2008; Rajalingham, Issa, Bashivan, Kar, Schmidt, & DiCarlo, 2018; Tripp, 2017; Yamins & DiCarlo, 2016). While there are many observed similarities—such as networks replicating some human visual illusions (Gomez-Villa, Martin, Vazquez-Corral, & Bertalmio, 2019; Ward, 2019; Watanabe, Kitaoka, Sakamoto, Yasugi, & Tanaka, 2018), there are also many striking dissimilarities, such as cases in which CNNs fail to replicate human behavior in simple tasks (Stabinger, Rodríguez-Sánchez, & Piater, 2016), react very differently from humans to slight changes in stimuli (Kurakin, Goodfellow, & Bengio, 2017; Nguyen, Yosinski, & Clune, 2015; Sharif, Bhagavatula, Bauer, & Reiter, 2016; Szegedy, Zaremba, Sutskever, Bruna, Erhan, Goodfellow, & Fergus, 2014), and show weaker performance than humans in generalizing across different forms of image degradation (Geirhos, Janssen, Schütt, Rauber, Bethge, & Wichmann, 2017; Geirhos, Schütt, Medina Temme, Bethge, Rauber, & Wichmann, 2018). It is important to note that merely finding comparable overall performance to humans in a given task is a weak basis for claiming equivalence, as there is a potentially infinite number of different models, with different architectures and internal representations, that could yield equivalent performance. Even comparisons based on correlations in responses across randomly chosen test images will tend to overestimate similarities between humans and models. This is because, if both humans and models perform well at the task, they will tend to give similar responses to most stimuli. Because they get the answer correct in most cases, they will necessarily correlate strongly. Yet, these correlations would simply indicate that both systems perform the task well. The shared variance would be driven by the ground truth, not necessarily by inherent similarities in the way they arrive at their responses. As a result, comparisons based on random stimuli generally do not distinguish between different computations that achieve equivalently good performance.

In order to reveal the computations that are specific to the human visual system, it is therefore necessary to decouple (i.e., decorrelate) human perception from the ground truth. We need images for which humans make mistakes to provide a source of variation in performance that is independent from the ground truth, which can indicate human-specific computations. This is challenging as human perceptual errors are relatively rare. Nevertheless, here, we identified a set of images that consistently yield misperceptions, allowing us to test which models predict the specific patterns in human perception. Using these images, we then varied the architecture of the CNNs to gain insights into which levels of computation are necessary and sufficient for reproducing human judgments. We systematically varied the depth (number of layers) of the networks and, for each depth, searched architectural and learning hyperparameters to identify networks that best matched humans. We reasoned that, if human gloss judgments are driven by sophisticated high-level representations, then deep (i.e., many-layered) networks would be required to reproduce human performance. In contrast, if human judgments are based on relatively simple image cues, shallow networks might be sufficient.

Broadly speaking, there are two ways we use the term “complexity.” One refers to stimulus characteristics. This could in principle be quantified in terms of information theoretic ideas, such as entropy. However, here we use the term more loosely, in the sense of an everyday intuition about highly structured images. For example, an image of glossy surface in a natural environment containing varied and structured patterns of specular highlights, shading gradients, and shadows is intuitively “more complex” than one that contains only smooth gradients or uniform random noise (even if the entropy of the latter is actually higher than in the natural image). Our other usage of the term refers to the sophistication of computations. Simple image measurements, such as first-order pixel statistics are, in an intuitive sense, “less complex” than computations that involve multiple stages of operations, which pool and select information across the image in conditional or nonlinear ways to determine distal surface properties from the image. This usage is also related to stimulus complexity, as complex stimuli tend to require more sophisticated computations. Again, the term is used loosely to indicate an intuitive sense of the degree of sophistication, such as the number of layers in a CNN and the number of nonlinearities in a network required to achieve a given response.

In a second approach we also used deep convolutional generative adversarial networks (DCGANs) (Goodfellow et al., 2014; Radford, Metz, & Chintala, 2015) to generate new images with ambiguous material appearance after training them on our high- or low-gloss image dataset. DCGANs consist of two networks—a generator and a discriminator—that are trained by working against each other. Specifically, the generator network synthesizes “fake” images, which the discriminator learns to distinguish from “authentic” training images (in our case, example renderings from the training set). The goal of the generator is to create images that the discriminator cannot identify as fakes. Both networks are trained simultaneously to improve at their respective tasks, causing the generator to create images that become progressively more difficult for the discriminator to distinguish from the training images. During training, the generator learns to synthesize images using new and different features, while the discriminator learns to identify these features to decide whether the image is generated or part of the training set. Again, we sought to identify at which network depths these images included features that humans can use to identify high- or low-gloss materials. We trained DCGANs of different network depths and showed the generated images to human observers to see at which depths observers can accurately distinguish recreations of high-gloss images from recreations of low-gloss images. Again, if human gloss classification is driven by simple, low-level image statistics, then relatively shallow DCGANs should be sufficient to evoke compelling gloss percepts in humans. In contrast, deeper DCGANs can reproduce more sophisticated image structures, yielding impressions of bounded objects with internally coherent surface and image structure. If humans require such cues, then the deeper DCGANs would be necessary to yield reliable gloss judgments.

## Methods

### Stimuli

To train our classifiers and test our observers we used 128 × 128-pixel computer renderings created with Radiance (Ward, 1994). We gathered a database of 1834 3D object meshes that included natural objects and manmade artifacts, as well as a set of 214 high-dynamic-range illumination maps (light probes). The light probes came from various sources, among them two scientific databases (Debevec et al., 1998; Adams et al., 2016; see the appendix for a list of all sources). We rendered random combinations of objects and illuminations, picking random viewing angles from a hemisphere above the object, with the object centered in the image. We rendered the object in a completely specular and a completely diffuse material for each scene and used a linear combination of these two to create different levels of gloss. For all experiments reported here, we only used two levels of gloss—high gloss (specular component × 0.98 + diffuse component × 0.02) and low gloss (specular component × 0.02 + diffuse component × 0.98). Because images of completely smooth diffuse materials could be easily distinguished from images of specular materials based on trivial cues such as overall brightness and contrast, we added textures to the diffuse component. The textures were created by mapping marbled distortions of randomly selected illumination maps onto the surface of the object. They were multiplied with the diffuse component. The overall formula for combining the components of our images was as follows:

specular image × specular weight + diffuse image × texture image × (1-specular weight) + (1-alpha map) × background

We discarded any images where the object covered less than 20% or more than 90% of the image. The total number of images was 149,922 (74,961 high gloss and 74,961 low gloss).

To train the DCGANs, we created another dataset with the same procedure and based on the same object meshes and illumination maps. The only difference was an increased viewing distance to ensure that the bounding box of the objects was completely contained in the viewing window. Initial tests showed that DCGANs produced images more resembling objects on a background rather than patches of material interspersed with patches of background when the objects in the training images were completely within the image boundaries. Again, we discarded images where less than 20% or more than 90% of pixels were covered by the object. This image set contained 187,630 images (93,815 high- and low-gloss images each).

#### Experiments with human observers

For all lab experiments with human observers, we presented 128 × 128-pixel images from our set of renderings on a black background. Participants were shown the images for as long as they took to respond. Before each experiment, participants saw 12 example stimuli at a resolution of 512 × 512 pixels (six low-gloss and six high-gloss images). These were not included as stimuli in the experiment. All observers had normal or corrected to normal vision and signed a consent form in accordance with the tenets of the Declaration of Helsinki (6th revision).

#### Random images experiment

Ten observers (all female; mean age ± *SD*, 26.1 ± 4.04 years) were shown 150 randomly selected images from each ground-truth material, one at a time. They were asked to classify the images as either high gloss or low gloss by pressing one of two different keys. There were four repetitions of the image set, each time in a different random order. Every 100 trials, participants were asked to take a short break. The experiment lasted between 1 and 1.5 hours.

#### Selecting a diagnostic image set

Responses to a randomly chosen image set are not well suited to assessing the similarity of a model to human observers. Because we expect both humans and at least some models to solve the task well, there will be a misleadingly high similarity in their responses to randomly chosen images. This similarity would be due to both sets of responses being similar to ground truth. Any model that solves the task better would therefore seem to be more similar to humans when it is actually only more similar to ground truth. We therefore selected a *diagnostic set* of images, in which human responses were decorrelated from ground truth. This diagnostic image set was selected in two steps. We first performed two series of prescreening experiments in the lab where observers would categorize images as either high- or low-gloss images. This resulted in 500 candidate images. The second step was an online crowd souring experiment in which participants judged the preselected images on a five-point rating scale from low to high gloss. Based on these responses we selected the final diagnostic set comprised of 60 images—30 from each ground-truth material—in which images from both materials were evenly distributed across three bins of perceived gloss.

#### Image prescreening for crowd sourcing

Over the course of five experiments, we used subject responses to select a set of 500 candidate images starting from 31,500 randomly selected images. Participants were shown images one at a time and were asked to classify them as high gloss or low gloss by pressing one of two keys. In addition, they could flag an image using the space bar if they found there was no recognizable object in the image. Every participant saw 1500 images. In the first round, we showed 15,000 images selected randomly from the overall set, divided among 10 subjects (eight female, two male; mean age ± *SD*, 24.8 ± 4.8 years), so each subject saw 1500 images (750 from each ground-truth material), and each image was judged by one subject. For the second round, we removed all images that were flagged as unrecognizable. We selected all of the remaining images that were classified incorrectly (587 low-gloss and 1817 high-gloss images) plus correctly judged images to total 2250 images from each ground truth category (1663 and 433 correctly judged low- and high-gloss images, respectively). These were judged by 15 participants (12 female, three male; mean age ± *SD*, 23.7 ± 3.8 years). Again, every participant saw 750 images from each ground-truth material, resulting in five judgments per image. These results were combined with the classifications of these images from the first round. From these results—six binary judgments on each image—we divided the images from each ground truth into seven bins according to the mean responses. For ground-truth high-gloss images, we picked 750 images—107 from each bin and 108 from the most incorrectly judged bin. For ground-truth low-gloss images there were not enough images in each bin to pick the same amount. Where this was the case, we picked all images from that bin and added the difference between the actual bin size and the target number of images to the target number for the next bin. We performed this procedure starting with the bin of most incorrectly judged images. The resulting set of 1500 images was then judged again by four participants (three female, one male; mean age ± *SD*, 22.5 ± 2.1 years), which resulted in 10 classifications per image after combining these results with those of the first two rounds.

Because the number of incorrectly perceived high-gloss images was much larger than that of low-gloss images, we repeated the search progress. This time we tested in two stages. In the first stage, we showed 16,500 images (12,000 high gloss and 4500 low gloss) to 16 subjects (14 female, two male; mean age ± *SD*, 23.8 ± 3.2 years)—1500 images each (750 low gloss and 750 high gloss), resulting in one classification response per low-gloss image. High-gloss images were included to balance the stimulus set, but the data were not used to identify candidate images. These were shown to several subjects, whereas low-gloss images were seen by only one subject each. For the second stage, we again removed all images that were flagged as unrecognizable and from the remainder took 750 low-gloss and 750 high-gloss images (favoring incorrectly judged low-gloss images) and tested nine more subjects (six female, three male; mean age ± *SD*, 23.9 ± 2.9 years) on these 1500 images, resulting in 10 binary judgments for each low-gloss image. We did not use the high-gloss images from this experiment because we already had enough to fill our diagnostic set from the first set of experiments.

The images from the final stage of the first set of experiments and the low-gloss images from the final stage of the second set of experiments were combined and divided into five bins ranging from “seen as low gloss” to “seen as high gloss.” We picked 50 images from each ground-truth material per bin, except for the most “seen as high gloss” bin, where there was only one ground-truth low-gloss image. We filled this bin up with ground-truth high-gloss images. These 500 candidate images were the set we used in the crowd sourcing experiment. These images and the images of the same scenes from the other material category were withheld from training the classifiers, leaving 148,922 images for training and validation.

#### Online crowd sourcing experiment

**Figure 1.**
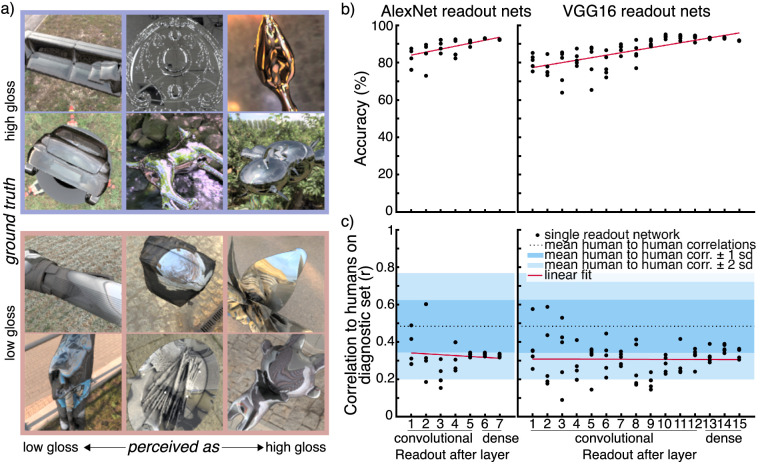
(a) Example images from the diagnostic image set. Images are sorted in columns according to increasing perceived gloss from left to right. Images in the top two rows were rendered in a high-gloss material, and images in the bottom two rows were rendered in a low-gloss textured material. (b) Classification accuracy of read-out networks from AlexNet and VGG16 (percent correct). For every read-out network, we trained five instances from random initialization. (c) Correlation of read-out networks to humans on the diagnostic image set (*r*). The dotted line shows the mean of correlation between individual human observers and the mean of the remaining observers. The blue areas show the first and second standard deviations.

For our crowd sourcing experiment, we recruited participants through an online platform, Clickworker. Instructions were shown in German and English at the same time, and participants were recruited to be between 18 and 60 years old. Ninety-nine people participated and judged the 500 images that resulted from our image prescreening experiments. Each participant was shown the same high-resolution example images we used in our lab experiments and then judged each image from our test set on a five-point rating scale from low gloss to high gloss. We included two photographs—one of a sandcastle and one of a silver teapot—as catch trials at the end of the experiment. Participant data were excluded if their datasets included too many or too few trials (resulting from using the back and forward buttons on their web browser) or if they failed to judge the teapot in either of the two highest gloss categories or the sandcastle in either of the two lowest gloss categories. Response data from 35 participants was excluded based on these criteria, leaving 64 participant responses. We divided the 500 candidate images into five bins based on mean gloss ratings of the 64 subjects. The diagnostic set had to be balanced across these bins and was therefore limited by the number of low-gloss images in the highest gloss bin (0) and in the second highest gloss bin (10). We chose 10 images from the three central bins for both materials (randomly for bins that contained more than 10 images) to make up the diagnostic image set. Example images are shown in Figure 1a.

### Classifiers

#### Experiments with diagnostic image set

As an initial test of CNN performance on our task and similarity of responses to humans, we applied readout training to the AlexNet (Krizhevsky, Sutskever, & Hinton, 2012) and VGG16 (Simonyan & Zisserman, 2015) networks. These networks were pretrained on the ImageNet object recognition task. We took the networks up to a certain layer and added a linear output layer (or dense layer), which we trained to perform our classification task, leaving the rest of the network unchanged. We did this for each convolutional and dense layer in each of the architectures, taking the readout after the subsequent pooling and rectified linear unit layers (or just before the next convolutional or dense layers). For each network and each readout layer, we trained five instances of the classifier, each time with a random initialization of the final dense layer. Models were trained on 90% of our images (134,030), and 10% of our images (14,892, balanced for both materials) were randomly picked and withheld as a validation set. Loss on the validation set was calculated every 100 training steps. Training would stop if there was no improvement for five consecutive validation steps. We chose this criterion rather than a fixed training duration to balance training for different architectures with different numbers of parameters.

**Figure 2.**
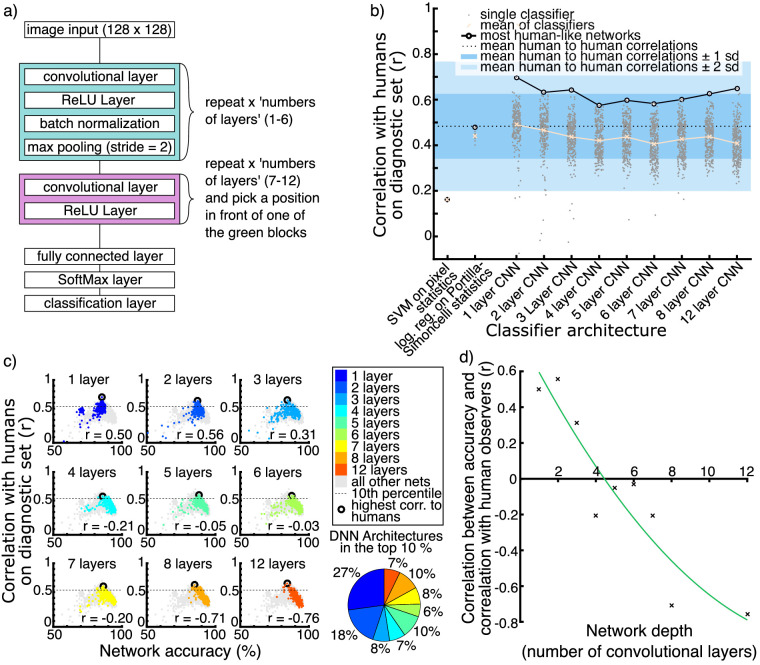
(a) General architecture of the CNNs in our Bayesian search. (b) Overview of the performance of linear classifiers and CNNs from the Bayesian search. Performance is plotted in terms of correlation to human observers on the diagnostic image set (*r*). For every linear classifier, we trained 20 instances. For every CNN depth, we trained 300 networks using a Bayesian search algorithm to optimize training parameters, as well as the number and size of convolutional filters within each layer. (c) Accuracy of each network plotted against correlation to humans on the diagnostic set for each depth of CNN. Gray points show all networks, and colored points show one network depth each. Correlation coefficients are shown. Bottom right shows the proportion of different CNN depths in the 10% of CNNs that correlated highest with humans. (d) Correlation coefficients from (c) plotted against network depth, showing a trend from positive correlations for shallow networks to negative correlations for deep networks.

To investigate how well a CNN trained from random initial weights could model human gloss perception, we wanted to train and test networks that span a large space of possible hyperparameter values. To train and test such a large number of networks we decided on a general architecture, as shown in Figure 2a. We also picked a number of hyperparameters to optimize. These were parameters of both the architecture (size and number of filters in each convolutional layer) and the training (learning rate, learning momentum, L2 regularization). For networks up to six convolutional layers, we included a max-pooling layer with a stride of two after each convolutional layer. For more than six layers, the image size became too small, so we added any further layers without subsequent pooling. In addition, we defined hyperparameters that allowed the search algorithm to change the position of these convolutional layers without pooling within the network.

We varied the hyperparameters by letting a Bayesian search algorithm search for those parameters which—after training—would result in a network with a high correlation with human observers on our diagnostic image set. Networks were trained on 90% of our images (134,030). Again, the training progress was monitored by calculating the accuracy and loss on a validation set every 100 training iterations. The validation set consisted of 10% of the rendered images (14,892) that were picked at random, balanced for both materials, and withheld from training. If there was no improvement in validation loss for five consecutive validation steps, the training would stop. The trained network was then tested on the diagnostic image set and its responses correlated with the mean judgments of human observers. The Bayesian search program used this correlation as the objective to be maximized. A Bayesian search approach to hyperparameter optimization has been shown to be effective in finding hyperparameter settings that yield a well-performing network (Snoek, Larochelle, & Adams, 2012). Here we use an optimization approach to look for those combinations of hyperparameters that cause a network after training to correlate highly with human observers.

In addition to these CNNs, we trained two linear models using hand-engineered features: SVM using pixel statistics and logistic regression using dimensionally reduced Portilla–Simoncelli color texture statistics (Portilla & Simoncelli, 2000). The pixel statistics we used were the mean, variance, skewness, and kurtosis of pixel luminance and saturation histograms. To the Portilla–Simoncelli statistics we applied principal component analysis to reduce the dimensionality from 3381 to 817 dimensions, which explained over 99% of the variance in our images captured by the 3381 parameters. Fifty-eight images were excluded from the training data (29 high-gloss images and renderings of the same scenes with the low-gloss material) for causing errors in the Portilla–Simoncelli color texture analysis, leaving 148,864 images. These classifiers were each trained 20 times by splitting our image set in half, training one network on one half and testing it on the other, and vice versa. This resulted in 10 predictions for each image from these classifiers.

#### Experiments with manipulated specular components

**Figure 3.**
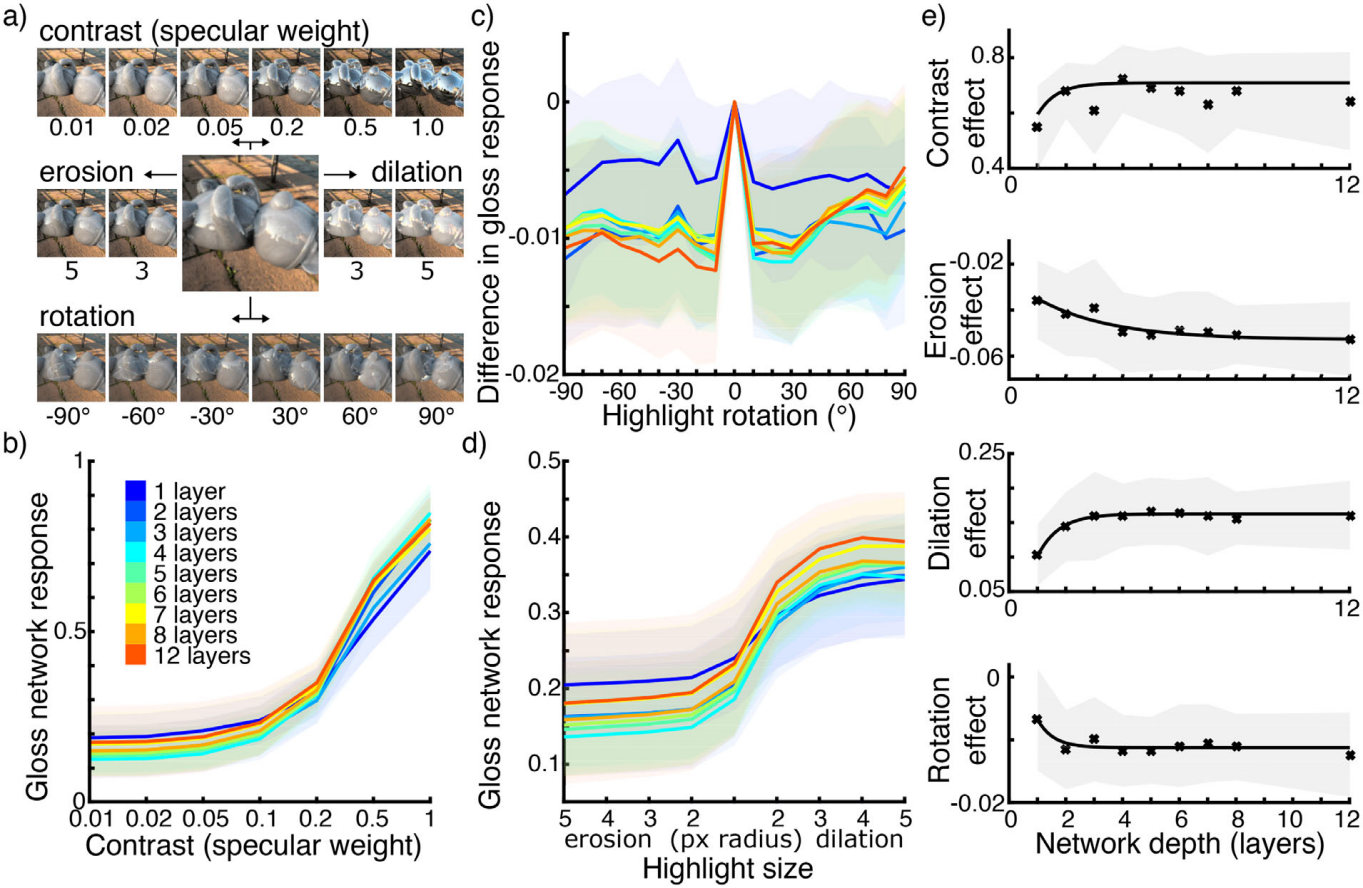
(a) Examples of gloss manipulations. The larger central image shows an image with unmanipulated specular component and a specular weight of 0.1. The top row shows manipulations of contrast by changing the specular weight. The middle row shows manipulations of the size of highlights by erosion and dilation. The bottom row shows examples of images with rotated specular components. (b) Mean responses of networks of different depths to images with different gloss contrasts (specular weights). Shaded areas show standard deviations. (c) Mean response differences for networks of different depths to images with rotated highlights. Note that the *y*-axis shows mean response differences rather than absolute responses, because network responses for each image are compared to a parallel image with specular components that are not rotated from the original but corrected for coverage according to the overlap in rotated images (see Methods for details). Shaded areas show standard deviations. (d) Mean responses of networks of different depths to images with differently sized highlights (through erosion and dilation). Shaded areas show standard deviations. (e) Mean effect sizes of gloss contrast, erosion, dilation, and rotation for networks of different depths. Shaded areas show standard deviations per network depth.

To test the reactions of our CNN models to factors that have been shown to influence human gloss perception (Anderson & Kim, 2009; Marlow et al., 2012) we prepared a test set of images for which we manipulated the specular component. See Figure 3a for examples. Specifically, we manipulated the *contrast* of highlights by changing the relative weight of the specular component (the levels we used were 0.01, 0.02, 0.05, 0.1, 0.2, 0.5, and 1.0), the *size* of highlights by applying erosion and dilation to the specular component (with radii from 2 to 5 pixels each), and the *orientation* of highlights by rotating the specular component of the images in steps of 10° up to 90° in both directions. For size and orientation manipulations, we chose an intermediate specular weight of 0.1. The training set contained images with specular weights of 0.02 and 0.98. We chose an intermediate level for these manipulated images, expecting intermediate responses for the unmanipulated images, so there would be no ceiling effects limiting network responses in either direction. For the orientation manipulations, we used an alpha channel to limit the specular component to the area that overlapped with the diffuse-textured component. To control for this reduced area of the specular component, we rendered parallel images to go with each rotated image, containing reflections at the correct orientation but cut to the same shape as was caused by the rotation. We applied all manipulations to 120 images, which were generated according to the same principles as the original training set but not included in the training or test image sets.

### DCGAN architecture

As a starting point for our DCGAN architecture, we used the architecture described in Radford et al. (2015). We added a fifth convolutional layer, because the original DCGAN was designed to generate 64 × 64-pixel images. From there we created architectures for shallower networks based on the following principles:

• Image resolution doubles between deconvolutional layers in the generator network and is halved between convolutional layers in the discriminator.
• Processing depth (number of filters) decreases by half for later deconvolutional layers and doubles for later convolutional layers.
• For shallower networks, we would skip processing at lower resolution. For a five-layer generator network, deconvolutional processing starts on a 4 × 4-pixel representation, for a four-layer generator at 8 × 8 pixels, for a three-layer generator at 16 × 16 pixels, etc.
• The latent space was the same for all network depths (100 × 1).

An overview of the resulting architectures can be seen in Supplementary Figure S2. We trained two instances of each architecture—one on low-gloss renderings and one on high-gloss renderings. DCGANs were trained using the MatConvNet toolbox for MATLAB (MathWorks, Natick, MA) (Vedaldi & Lenc, 2015). Because DCGANs have no objective function that captures model performance and image quality (Salimans, Goodfellow, Zaremba, Cheung, Radford, & Chen, 2016), we included an assessment of image realism in our experiment with human observers (see below).

### Human responses to DCGAN images

**Figure 4.**
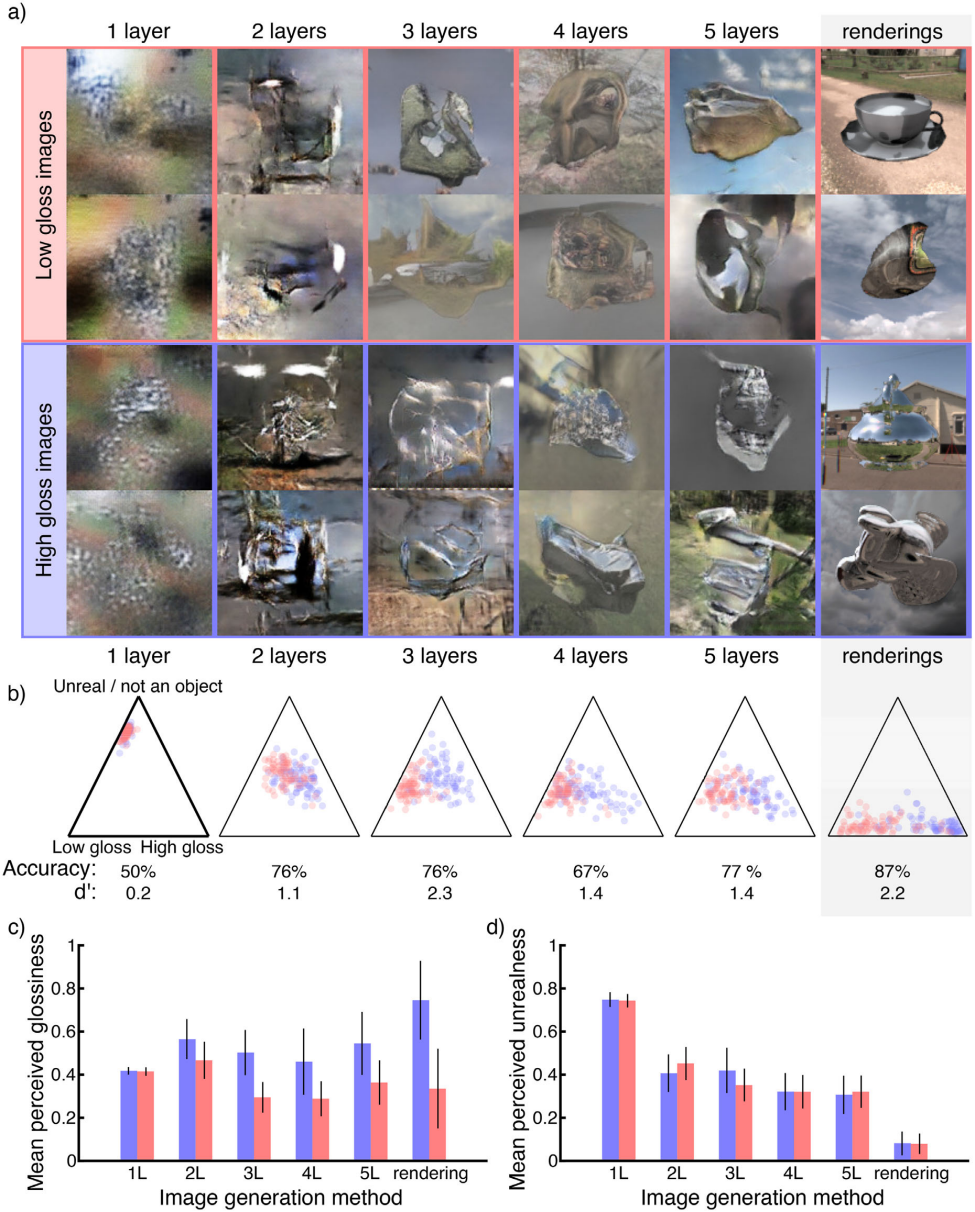
(a) Example images generated by DCGANs of different depths and renderings in the right-most column. The top two rows show images from networks that were trained on low-gloss textured images, and the bottom two rows show images by networks trained on high-gloss images. (b) Mean ratings from 15 human observers. The left-most triangle shows the labels that were displayed during the experiment. (c) Mean and standard deviation of gloss ratings for each network depth. (d) Mean and standard deviation of unrealness/not an object ratings per network depth.

We selected 75 images generated from each of our 10 DCGANs, resulting in 150 images from each network architecture, half of which were based on low-gloss and half on high-gloss renderings. In addition, we added 75 randomly picked low-gloss and 75 high-gloss images to the stimulus set, making a total of 900 images. See Figure 4a for example images.

Fifteen subjects (three female, 12 male; mean age ± *SD*, 24.2 ± 4.0 years) were shown these images one at a time and were asked to respond on a triangular rating field. The triangle corners were labeled “low gloss” and “high gloss” along the horizontal bottom edge and “unreal/not an object” on the top corner. Figure 4b shows the rating scale. Observers moved the position of the cursor within the rating field with the mouse. The experiment lasted between 1 and 1.5 hours.

## Results and discussion

### Human performance on random and diagnostic images

We created a dataset of 74,961 scenes, showing a familiar object under image-based illumination. Each scene was rendered once with the object made of a mirror-like (high gloss) material and once with the object made of a near-matte textured (low gloss) material, yielding a total of 149,922 images.

Human observers were mostly able to discriminate between our two material categories. We asked 10 participants (all female; mean age ± *SD*, 26.1 ± 4.0 years) to classify 300 randomly selected images based on their glossiness. There were 150 images from each material category, and every observer saw each image four times. On average, human observers judged 87.6% of images correctly (*SD* = 4.6%).

Responses to such a randomly chosen image set are not a sufficient criterion to evaluate how well a model replicates human perceptual judgments. It is not enough for a model to make the same number of correct or wrong decisions as humans, but rather a good model should also make correct or wrong decisions on the same images that humans do. On a randomly chosen image set, humans perform well above chance, and their responses are highly correlated with ground truth. Any model that solves the task well will therefore also correlate highly with human observers. Thus, to decorrelate model accuracy from similarity to humans we assembled a diagnostic set of images in which mean human judgments were decorrelated from ground truth. In a series of lab experiments, we showed a total of 31,500 images to 54 participants (43 female, 11 male; mean age ± *SD*, 23.9 ± 3.5 years) in a binary classification task and used their responses to identify 500 candidate images that frequently yielded errors. The experiments were conducted in several rounds, where we used subjects’ responses to narrow down the set of candidate images for later rounds. Some images were seen by only one observer, but the final candidate images were seen by 10 observers (see Methods and Supplementary Figure S1 for details). From these candidate images, we selected 500 images that we showed to 99 participants (20–64 years of age; no gender information available) in a crowd sourcing experiment to judge each image on a five-point rating scale from high to low gloss. We excluded data from 35 participants based on double trials or skipped trials (resulting from using the back or forward buttons in their web browsers) or failing at least one of two catch trials at the end of the experiment (see also Methods). From the ratings of the remaining 64 participants, we identified the 60 final images that made up our diagnostic image set. The diagnostic set contained 30 images from each of the two reflectance categories. These were selected so that the mean responses across crowd sourcing participants would classify an equal number of images wrongly, correctly, and half-way between high and low gloss. This dataset allowed us to test to what extent a model makes perceptual decisions similar to those of humans, independently of the accuracy of the model. Thus, on this diagnostic image set, human performance was by definition chance (53.3% accuracy of mean human responses). Correlation between mean human response and ground truth was *r* = 0.13 and *p* = 0.32. Example images from the set are shown in Figure 1a.

### Read-out networks

As a pilot experiment to test different model complexities, we looked at read-out networks of two well-researched CNN architectures, AlexNet (Krizhevsky et al., 2012) and VGG16 (Simonyan & Zisserman, 2015), which had been trained on the ImageNet object recognition task. At the time this study was conceived, VGG16 was state of the art in imitating the architecture of the human ventral stream. We used two architectures to ensure that observations we made about readout networks would generalize between source networks and were not specific to either one. We trained a linear classifier on representations at different stages throughout both networks to perform the high-gloss versus low-gloss classification task. For each layer from which we took read-out features, we trained five instances of the linear classifier. The performance of these networks in terms of accuracy and their correlation to humans on the diagnostic image set is shown in Figures 1b and 1c, respectively. There are two notable trends: The accuracy improved for read-out networks from later layers (Pearson correlation between mean accuracy and readout layer for AlexNet: *r* = 0.933 and *p* = 0.002; for VGG16, *r* = 0.891 and *p* < 0.001), and read-out networks based on VGG16 representations from earlier layers showed more variance in their correlations to human observers between instances (Pearson correlation between variance in correlation to humans and readout layer, *r* = −0.724 and *p* = 0.002). Read-out networks based on AlexNet representations showed a non-significant correlation in the same direction (*r* = –0.593, *p* = 0.160). Yet, crucially, we also found that single instances of read-out networks with the highest correlation to humans for both AlexNet and VGG16 were trained on representations of early layers (the maximal correlations for both were achieved from second layer representations). This led us to expect that, for CNNs trained from random initial weights, we can find exemplars of shallow networks that correlate well with human observers. Early layer features of object recognition CNNs tend to capture more localized spatial regularities than later layers and have been associated with textures (Zhou, Bau, Oliva, & Torralba, 2018). This could also mean that texture statistics may already provide a basis for human-like gloss discrimination—at least for the types of images in our training and test sets—even if such image properties are insufficient to account for all phenomena in gloss perception (Anderson & Kim, 2009; Kim et al., 2011; Marlow & Anderson, 2015; Marlow et al., 2011; Marlow et al., 2015).

### CNNs and linear classifiers

For a detailed sampling of CNN architectures that had not previously been trained on other data, we conducted a Bayesian search, training 2700 networks from random initial weights to identify the ground truth class (low vs. high gloss) of 134,030 images from the rendered training set. We used the Bayesian search algorithm to optimize hyperparameters of the training procedure and the architecture of networks with 1, 2, 3, 4, 5, 6, 7, 8, and 12 convolutional layers, training 300 models for each network depth. The Bayesian search optimized model correlation with human responses on the diagnostic image set. Models were trained on 90% of our image set, with 10% (14,892 images) being selected at random and withheld as a test set. The images in the diagnostic set were always withheld from training. For our general model architecture, see Figure 2a; for details on the training, see Methods.

We also trained two hand-engineered classifier models. One was a logistic regression trained on features we derived from a texture model of human mid-level vision (Portilla & Simoncelli, 2000; see also Methods for details). Finally, we trained a SVM on eight pixel statistics (mean, variance, skewness and kurtosis of the pixel luminance and saturation histograms) to differentiate high-gloss from low-gloss images. We expected linear models trained on pixel statistics to insufficiently model human responses. At the same time, this is a useful benchmark to test more complex models against and to ensure that the discrimination task provided by our stimuli is not trivial. For training these hand-engineered classifiers, we split our image set in half, training one classifier on each half and testing it on the other (twofold cross-validation). We repeated this 10 times for each model.

An overview of how all of the CNNs and the two linear classifiers correlated with human observers on the diagnostic set can be seen in Figure 2b. We excluded all “dead” networks (i.e., networks that resulted in a failed training or constant predictions to all stimuli). These were 61 in total (27, 23, 2, 0, 1, 1, 3, 2, 2 for 1-, 2-, 3-, 4-, 5-, 6-, 7-, 8-, and 12-layer networks, respectively). We compared these correlations against the distribution of correlations of individual human observers with the mean of the remaining 63 human observers. The mean, as well as the first and second standard deviations of this distribution, can be seen in the dotted line and shaded regions in Figure 2b. The SVM on pixel statistics clearly correlates with humans much less than the other classifiers. The logistic regression on Portilla–Simoncelli statistics is close to the mean of human-to-human correlations. Interestingly, there are examples of CNNs from all depths that correlate well with humans, and there is no obvious trend or difference between the depth groups.

On average, individual human responses to the diagnostic stimulus set correlated at 0.47 with the mean of the remaining observers, explaining only 22% of the variance in the mean responses. This means that human responses are quite idiosyncratic for the diagnostic stimulus set. The logistic regression based on Portilla–Simoncelli statistics reached a similar correlation to the mean human response vector. However, the best of the CNN models correlated to 0.7 with the human mean, explaining 50% of the variance. The most human-like CNNs therefore explain more of the central tendency in human responses than the responses of most individual human observers do. This could reflect the fact that the human visual system has internal noise (e.g., Pelli & Farell, 1999), whereas CNNs do not. This is an interesting topic for speculation, but the current data do not allow for strong conclusions about the nature of noise within the data for individual observers, so further research on the origins of individual differences in gloss perception is necessary.

An ANOVA revealed a main effect of network depths, *F*(8, 2638) = 34.4, *p* < 0.001, indicating that, on average, networks of different depths correlated differently to human observers. One-layer networks had the highest mean correlation (mean *r* = 0.49; for the other depth groups, mean *r* ranged from 0.41 to 0.47). However, our Bayesian search algorithm was not designed to characterize the mean similarity to humans for an entire hyperparameter space. Rather, it searched for those settings and individual networks that correlated highly with humans, choosing new settings to investigate particularly interesting regions of the hyperparameter space, rather than sampling the space in a grid-like fashion. Looking at the top 10% of most human-like classifiers we find CNNs from all depths (see Figure 2c), indicating that single exemplars from different network depths may result in high correlations to humans. The most human-like of all networks was a one-layer network, and indeed 27% of the top 10% most-human networks were only one layer deep, followed by a further 18% of two-layer networks. This suggests that, when using the Bayesian search approach we applied, it is easier to find relatively shallow networks that resemble humans than deeper ones. This may reflect a use of relatively simple cues by humans, although it may also be related to the much smaller parameter space to be searched for shallow networks.

Having decorrelated human responses from ground truth, it is important to look at the performance of models as well as their similarity to humans. We sought to answer whether there is a systematic relationship between performance on the objective function the networks are trained on (i.e., accuracy at gloss classification) and their tendency to reproduce human patterns of gloss judgments. We therefore investigated how similarity to human responses and model performance are connected and whether those CNNs that responded most like humans are outliers in terms of their performance or show typical levels of performance for their network depth. To do this, we looked at the relationship between network accuracy (on the randomly picked 10% of images that were kept from training and used as a validation set) and the correlation coefficients to humans on the diagnostic set. Overall, these two factors barely correlate (*r* = −0.1, *p* < 0.001), confirming that, when using the diagnostic image set, human-specific response characteristics can be measured independently of overall performance. Yet, looking at the groups of networks with the same numbers of layers (see Figure 2c) revealed a clear trend: For shallower networks, there is a positive correlation between accuracy and correlation with humans, meaning those exemplars that correlate well with humans are also the ones that perform better within the range of possible networks. For deeper networks, there is a negative correlation. In other words, of the deep networks, the ones that correlate well with humans performed the task badly relative to other networks of the same depth. This also indicates that there is an intermediate range of network depths where there is little or no correlation between accuracy and correlation with humans and where a high correlation with humans is more typical. Figure 2d shows the correlation coefficients of the plots in Figure 2c as a function of network depth, showing the trend of decreasing correlation with network depth. A quadratic fit to the trend reveals an intersection with zero correlation at a depth of approximately four or five convolutional layers. Taken together, these analyses suggest that relatively shallow networks tend to be those that, typically and independently of their performance on the training objective, tend to correlate most closely with humans.

Our results suggest that the task of distinguishing high-gloss from low-gloss textured materials in a similar way to humans does not require the complexity of very deep convolutional networks. Indeed, even a linear classifier using texture statistics can match human perceptual judgments on the task at the level of human-to-human mean correlations. However, CNNs are able to explain mean human responses even better. There is no improvement of deeper networks over shallower ones, despite their increased objective accuracy at the task. Analysis suggests that networks with approximately four or five convolutional layers tend to typically correlate well with humans.

To further investigate similarities in network responses to human observers, we rendered a set of images with manipulations to the specular component that have been shown to influence human gloss perception (Anderson & Kim, 2009; Marlow et al., 2012); see also Methods for a detailed description and Figure 3a for examples. These manipulations were changing the *contrast* of specular reflections by changing the specular weight, changing the *size* of highlights by eroding or dilating the specular component, and *rotating* the specular component. The mean responses from networks in each depth group for 120 images with these manipulations are shown in Figures 3b to 3d. Networks appear to be very sensitive to specular contrast, predicting images with higher contrast reflections to be glossier. This is not surprising, as specular weight was the primary manipulation in the training data. Networks also react to highlight size, predicting images with eroded highlights to be less glossy and images with dilated highlights to be glossier. Rotations of highlights appeared to have little influence on network responses. On average, networks predicted images with rotated highlights to be less glossy than images with correctly oriented highlights. This effect did not appear to increase with the angle of rotation.

To compare the effects these manipulations had on network responses, we calculated an effect size for each manipulation and compared this among networks. For the contrast manipulation, we took as an effect size the differences in responses between the highest and lowest specular weight conditions we tested (1.0 and 0.01). For size manipulations, we calculated two separate effect sizes for erosion and dilation. For each, we took the differences in responses between images containing unmanipulated highlights and images with the most dilated or most eroded highlights we tested (both with a radius of 5 pixels). For highlight rotations, we took the difference in responses between images with rotated reflections and parallel images with non-rotated reflections for the orientation with the largest effect per depth group. Figure 3e shows these effect sizes for each depth group of networks. For all four manipulations, the effect was smaller for very shallow networks, increasing with network depth but also reaching a maximum. This maximum appeared to be reached after network depths of three to six layers.

Our CNNs showed different degrees of sensitivity to different manipulations to the specular components of images. It is not surprising that networks reacted strongly to changing levels of specular contrast or specular weight. This is the primary difference between the two material classes in the training set. It is, however, interesting to see that intermediate specular weights also caused intermediate responses and that on average responses ordinally matched specular weights. Size of highlights also changed network responses, with more eroded highlights causing lower gloss predictions and more dilated highlights causing higher gloss predictions on average. Rotations of the specular component appear on average to have caused networks to judge images as less glossy. This effect was very small, however. A possible explanation lies in the training data and task. The training data contains only high-gloss mirror-like material or low-gloss material showing shading and texture and some specular highlights. Although congruence between shading and highlight components is a part of the typical appearance of the low-gloss material, learning this aspect of appearance is of little consequence for the network to improve at achieving the objective. Images containing both shading and specular highlights (and texture) are already at the low end of the learned gloss scale.

The effect sizes of highlight manipulations changed with network depth. All effects were smaller for very shallow networks and increased with network depth until they appeared to reach a maximum after about three to six layers. The directions of these effects are in line with what we would expect from human observers—higher specular contrast, larger highlights, and correct orientation all lead to higher gloss judgments on average. However, we cannot draw any conclusions about the absolute size of these effects. It seems likely that our training data and objective, representing only a limited part of gloss appearance and perception, provided only limited opportunity for learning some aspects of gloss perception. The leveling out of effect sizes with increasing network depth however seems to imply that, in so far as these features can be learned by networks in our training, they are maximally learned after about three to six layers. Deeper networks may learn more complex features, but they do not appear to be more sensitive to these manipulations. This is in line with our previous observation that it is more typical for networks of intermediate depths to correlate highly with humans and that further increased network complexity does not necessarily increase network similarity to human observers.

### Generative models

One limitation with using renderings to evaluate human gloss perception is that ambiguous stimuli are relatively rare. To increase the number of stimuli that could be diagnostic of human perception, in a second experiment we turned to DCGANs (Goodfellow et al., 2014; Radford et al., 2015) to generate images that share certain high-order statistical characteristics with renderings but which elicit a less distinctive surface appearance. This also allowed us to compare the necessary ingredients for networks to create images that appear glossy to humans with those required by classifier networks to match human judgments. Whereas in the previous experiments we searched for architectures of networks that classify low- and high-gloss images in a similar way that humans do, in this experiment we looked at different architectures of networks that generate images of low- and high-gloss materials that are distinguishable to human observers. For this purpose, we trained five DCGAN architectures to replicate low- and high-gloss images ranging from one to five convolutional layers. Of each architecture we trained two exemplars—one on high-gloss renderings and one on low-gloss renderings. By training the DCGANs separately on each class of images, we have ground-truth labels for which material is being recreated in each image.

From each DCGAN, we generated 75 images. In addition, we randomly picked 75 high-gloss and 75 low-gloss renderings from the image set we used for training the DCGANs. Overall, this gave us a set of 900 images (5 architectures × 2 image classes × 75 generated images + 75 × 2 renderings). We showed these to participants one image at a time in random order and asked them to rate the images within a triangular rating area. The labels of the three corners were “high gloss” and “low gloss” (along the horizontal axis) and “unreal/not an object” on the top corner. See Figure 4a for example stimuli.

Fifteen subjects (three female, 12 male; mean age ± *SD*, 24.2 ± 4.0 years) participated in this experiment. Their results are shown in Figures 4b to 4d. We separated subject responses into glossiness and realness—the horizontal and vertical components of their responses within the area of the triangle, respectively. We conducted a two-way repeated-measures ANOVA of glossiness responses. Mauchly's test indicated that the sphericity assumption was violated, χ^2^(65) = 218.4 and *p* < 0.001. Because Greenhouse–Geisser ε = 0.246, we report *p* corrected according to Greenhouse–Geisser. The ANOVA revealed two within-subject main effects: the image generation method, *F*(5, 70) = 8.2 and *p* = 0.003, and the image ground truth, *F*(1, 14) = 97.9 and *p* < 0.001. These imply that images from different generation methods (networks of different depths or renderings) are perceived differently in terms of their glossiness. Similarly, overall, observers could tell the low-gloss images and high-gloss images apart. The ANOVA also revealed an interaction term, *F*(5, 70) = 40.0 and *p* < 0.001, meaning that the difference between high- and low-gloss images changed between different image generation methods. To better understand this interaction, we conducted a series of comparisons of estimated marginal means of glossiness ratings of low- and high-gloss images for each image generation method separately. These comparisons revealed significant differences in glossiness ratings between low- and high-gloss images for all image generation methods except one-layer networks, *t*(14) = 0.93 and *p* = 0.368; for all others, *t*(14) = 5.61 or larger and all *p* < 0.001.

To quantify the difference between low- and high-gloss images from different generation methods, we looked at the classification accuracy. We define accuracy as the proportion of images rated on the correct half of the glossiness scale. These are shown in Figure 4b. In terms of accuracy, images from two-layer networks could already be discriminated as well as those from five-layer networks (76% vs. 77%). However, the main effect of image generation method on glossiness ratings indicates that the mean ratings across low- and high-gloss images differed among network depths, making the 50% criterion questionable. Visual inspection of Figure 4b shows that for networks of three to five layers the image ratings shifted overall toward the low-gloss end of the axis. As a measure of how well the glossiness ratings of the low- and high-gloss images were discriminable independent of a threshold criterion, we calculated a sensitivity index, *d*′, for each network depth. These were *d*′ = 0.17, 1.1, 2.3, 1.4, 1.4, and 2.2 for one- to five-layer DCGAN images and renderings, respectively. According to these values, images from two-layer networks were less discriminable than those from four- or five-layer networks. However, images from three-layer networks showed the same discriminability as renderings. An interesting observation is that, by visual inspection of Figures 4b and 4c, it appears that the perception of high-gloss images shifted for deeper DCGANs, whereas low-gloss images (red dots in the figure) remained mostly in the same position. This could indicate that high-gloss perception is more specific and that perception of low-gloss material can be achieved by a wider range of image quality and features.

We also performed a two-way repeated-measures ANOVA of “realness” responses. Mauchly's test again indicated violation of the sphericity assumption, χ^2^(65) = 276.6 and *p* < 0.001. We report *p* corrected according to Greenhouse–Geisser, as Greenhouse–Geisser's ε = 0.205. The ANOVA of realness responses revealed a within-subject main effect of image generation method, *F*(5, 70) = 34.1 and *p* < 0.001. A main effect of image ground truth was not significant, *F*(1, 14) = 0.1 and *p* = 0.808, indicating that low- and high-gloss images were not, overall, judged differently in their realism. An interaction was also significant, *F*(5, 70) = 4.9 and *p* = 0.022), meaning that for the different image generation methods realness differences between low- and high-gloss images varied. A series of comparisons of estimated marginal means of low- and high-gloss image realness for each image generation method separately revealed a difference only for images from two-layer networks, *t*(14) = 3.66 and *p* = 0.003. This indicates that, for all network architectures except two-layer networks, the quality of low- and high-gloss images in terms of realism was not perceived differently by observers. Together, these findings suggest that the lowest level texture-like statistical image structures that can be reproduced by one-layer DCGANs are insufficient to create distinct and realistic impressions of low- and high-gloss materials. Increasing depth led to more realistic and more distinct impressions, confirming that mid-level image organization factors play an important role in the perception of surfaces and their reflectance properties.

## General discussion

There has been a long-running debate about which level of processing or type of information observers rely on to identify surface gloss—relatively low-level image statistics (Boyadzhiev et al., 2015; Motoyoshi et al., 2007; Sawayama & Nishida, 2018) or more sophisticated mid-level representations that capture relationships between image features and 3D surface structure (Anderson & Kim, 2009; Kim et al., 2011; Marlow & Anderson, 2015; Marlow et al., 2011; Marlow et al., 2015). Yet, the classes of information relevant for gloss judgments could plausibly vary depending on the task. Here, we focused on one of the most basic gloss perception tasks: classifying a whole image as either high or low gloss. We asked observers to classify a diverse set of low-resolution renderings of textured low-gloss and untextured high-gloss surfaces. To our knowledge, this is one of the largest scale gloss perception experiments performed to date, spanning tens of thousands of images.

Overall, we found that observers were good but far from perfect at distinguishing them—providing ample possibilities to identify the human-specific patterns of responses. To gain insights into the kinds of cues and computations observers used, we identified a set of diagnostic images in which human responses were systematically decoupled from ground truth, which we then compared against a variety of models, based on different types of computation. While we could rule out simple intensity and saturation statistics, features from a well-known texture analysis/synthesis algorithm (Portilla & Simoncelli, 2000) could predict mean human judgments roughly as well as individual observers could, and a range of CNNs even better. Together, these findings hint that, although extremely simple image statistics cannot account for human gloss categorization, relatively straightforward mid-level image statistics, without any explicit representation of 3D structure, might be sufficient to account for human performance in this simple task. This raises the intriguing possibility that there might be a typical overall “look” of matte and glossy surfaces that can be captured by texture-like image statistics and which is sufficient for matte versus glossy decisions.

By systematically varying CNN architectures and hyperparameters, we sought to identify certain CNNs that more closely resembled human performance than others. However, we found that the extent to which the CNNs correlated with human judgments varied surprisingly little across changes in network depth. Although an ANOVA revealed a main effect of network depth, with one-layer networks correlating highest with humans on average, there are several points to be made against considering only the mean correlation to humans per network depth. The first is that the Bayesian search algorithm is not intended to investigate the mean correlation to humans. Rather, it searches the hyperparameter space for settings or a range of settings that maximize correlation to humans, as opposed to an evenly spaced grid search. Our data show that there are human-like networks for every depth we investigated.

Another point to be made about the mean correlations of depth groups is that we cannot discriminate between variance in model performance due to random initialization and variance due to changing hyperparameters. It is possible that random initialization causes more variance than changes in particular hyperparameters for our training set, objective function, and network architectures. We have also seen effects of this in our read-out networks, where the random initialization of only a final linear layer led to a wide range of correlations with humans.

We therefore use the analysis of network performance typicality shown in Figures 2c and 2d as summary statistics. We found that very deep networks that correlate highly with humans tended to perform relatively poorly in terms of classification accuracy. Shallow networks that responded in a very human-like way tended to perform relatively well. Networks of intermediate depths (four or five layers) typically showed human-like responses, independent of their accuracy. Although the most human-like single network we observed was a one-layer network, we suggest that the networks of intermediate depths are of particular interest in modeling human gloss perception, because they more typically respond similarly to humans, not just in outlier exemplars. It is also worth noting that the high prevalence of shallow networks in the top 10% most human-like networks may also be related to the size of the search space. Deeper networks have more hyperparameters and thus may require additional optimization to identify human-like exemplars. Taken together, the analyses suggest that very deep representations are not required to predict human-like gloss categorizations.

These results are supported by the responses by the CNNs to images with manipulated highlights. We showed images with changes in specular contrast, highlight size, and highlight orientation to all CNNs from our Bayesian search. Humans have been shown to be sensitive to similar manipulations (Anderson & Kim, 2009; Marlow et al., 2012). The results (Figure 3) show that networks are sensitive to these manipulations to different degrees. Although we cannot compare the magnitude of the effects among manipulation conditions, we can compare them among networks. On average, network responses shifted, as we would expect from human observers. Effect sizes in responses to all manipulations increased for very shallow networks and reached a maximum after a network depth of about three to six layers. Deeper networks showed no further increased sensitivity to these manipulations, indicating that intermediate networks learned these features as much as can be learned from our training data and task. This broadly agrees with our previous observation that intermediate CNN depths are sufficient to model human gloss perception.

Our experiment with DCGAN images showed that human observers were able to discriminate high-gloss from low-gloss images generated by two-layer networks. Three-layer networks were able to generate images that were essentially as discriminable as renderings to human observers. Although four- and five-layer DCGANs showed a decrease in discriminability, this cannot be easily explained by image quality as quantified by subjects’ judgments of image realism. Possibly the complex features enabled by the increased depth of the models do not contribute to gloss perception. The generator learns features (up to a certain limit) in order to replicate the image as well as the discriminator can identify, whereas the discriminator learns to identify features that the generator has not yet learned in order to discriminate generated images from training images. We found that, starting in two-layer networks and very much so in three-layer networks, the replicated features included at least some that human observers perceive as belonging to high- or low-gloss materials. Although this on its own does not tell us exactly which features are necessary and sufficient, it provides converging evidence that very shallow representations are insufficient to capture the structures on which humans rely, and very deep representations are not necessary.

Another interesting observation is that subjects can distinguish the glossiness of images even when they do not report perceiving an object in the image. This again hints that perception of gloss—at least at the level of simple binary classification—might be possible without perception of a coherent 3D surface. To fully evaluate this, it would be necessary to test shape perception for the DCGAN stimuli, which is an interesting avenue for future studies offered by these intriguingly ambiguous stimuli. Due to the processing differences between shallow and deeper networks, it could also mean that more local cues are sufficient to perceive glossiness than are necessary to perceive an object. This is also in line with the observation from our results with read-out networks, that those single instances of classifiers that best predicted human gloss responses were trained on features from early intermediate layers. AlexNet and VGG16, from which the features for our read-out networks were taken, were trained to recognize objects. Yet, the features that yielded the single most human-like read-out networks for the gloss perception task were also from an earlier stage of processing than those necessary for image recognition, suggesting again that gloss perception does not require object perception. Taken in sum, we believe our analyses provide convergent evidence that relatively low- to mid-level statistical image representations might be sufficient to account for human visual low-gloss versus high-gloss category decisions. Taking into consideration work on cortical representations (Freeman & Simoncelli, 2011; Hiramatsu, Goda, & Komatsu, 2011; Komatsu & Goda, 2018; Nishio, Goda, & Komatsu, 2012; Okazawa, Goda, & Komatsu, 2012; Sun, di Luca, Ban, Muryy, Fleming, & Welchman, 2016; Wada, Sakano, & Ando, 2014), it is interesting to speculate that ventral stream areas spanning V1 to V4 might be those most important for such judgments.

## Conclusions

Our work has narrowed down and identified some architectural conditions under which CNNs (as classifiers or as DCGANs) learn features that cause similar responses to humans or that allow humans to perceive generated images as high or low gloss. There is convergent evidence from our experiments that relatively shallow architectures are sufficient to model human gloss perception with a CNN. This is supported by results of read-out networks based on AlexNet and VGG16 representations, in which single instances of linear classifiers trained on representations at early stages of these networks showed the highest correlations to human observers. Correlating the accuracies of our CNNs to their correlation coefficients with human observers showed that CNNs of approximately four or five layers more typically correlated well with humans on our task than deeper networks. We also found that networks of about three to six layers reached a maximum mean sensitivity to manipulations of highlight contrast, size, and orientation. These intermediate network depths might be good candidates for further studies on modeling human gloss perception with supervised networks. Human ratings on images generated by different DCGAN architectures showed that generative networks with two convolutional layers were enough to recreate high- and low-gloss materials in a way that humans could tell them apart, whereas three convolutional layers were enough for human observers to distinguish as well as renderings. Linear classifiers using pixel intensity and saturation statistics were not enough to imitate human observers, and, although the correlation to humans of logistic regressions trained on texture statistics came close to mean human-to-human correlations, they were surpassed by CNNs. Overall, these results support the view of gloss classification as a computation of low- to mid-level vision.

## Supplementary Material

Supplement 1
