# Supplementary material for: Gloss perception: Searching for a deep neural network that behaves like humans
Source: J Vis. 2021 Nov 24;21(12):14. doi: 10.1167/jov.21.12.14 (PMC8626854; doi:10.1167/jov.21.12.14)
Supplement: Supplement 1 [file jovi-21-12-14_s001.pdf]

Appendix

Diagnostic image set selection process

Supplementary Figure 1

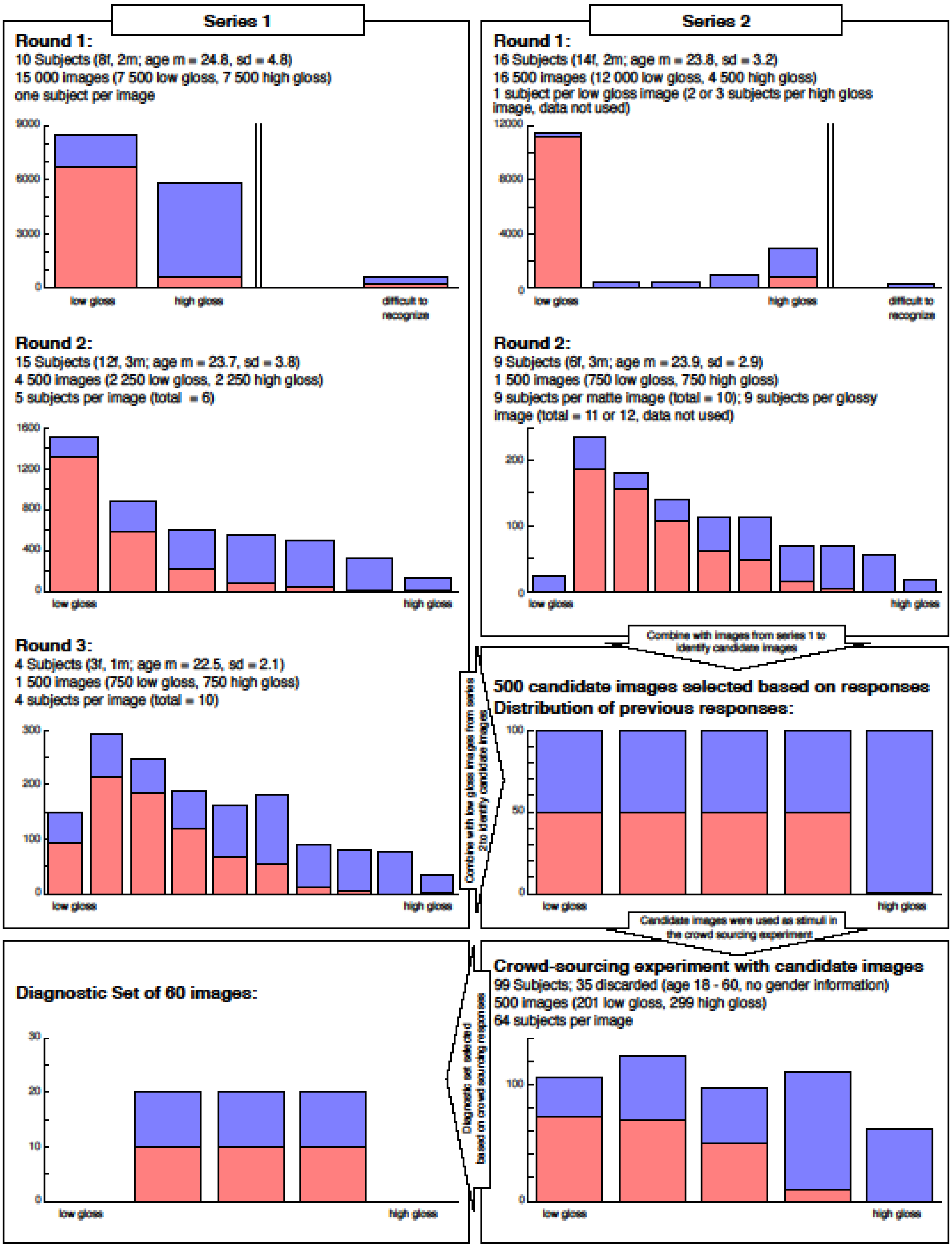

Supplementary Figure 1 - Legend

Figure S1:

The selection process of the diagnostic image set consisted of two series of experiments in our lab and one crowd sourcing experiment. See **Methods** for details. Blue indicates ground truth high gloss images, red indicates ground truth low gloss images, bars are stacked. Every graph shows the mean of subjects' binary high-gloss / low-gloss responses. **Top left box:** the first series of experiments started with 15 000 images, which we narrowed down to 1 500 images over the course of two experiments (top and middle graphs). The final 1 500 images were rated by 10 observers each (bottom graph). **Top right box:** The second series of experiments started with 16 500 images, of which only the 12 000 low gloss images were of interest to us, as the first series had already yielded a sufficient number of misperceived and ambiguous high-gloss images. Over two experiments these were narrowed down to 750 low-gloss images. **Center right box:** We selected 500 candidate images based on subjects' responses in the two series of screening experiments. **Bottom right box:** Responses to 500 candidate images from an online crowd-sourcing experiment. **Bottom left box:** The distribution of crowd sourcing responses for the 60 images in our diagnostic set

DCGAN architectures

Supplementary Figure 2

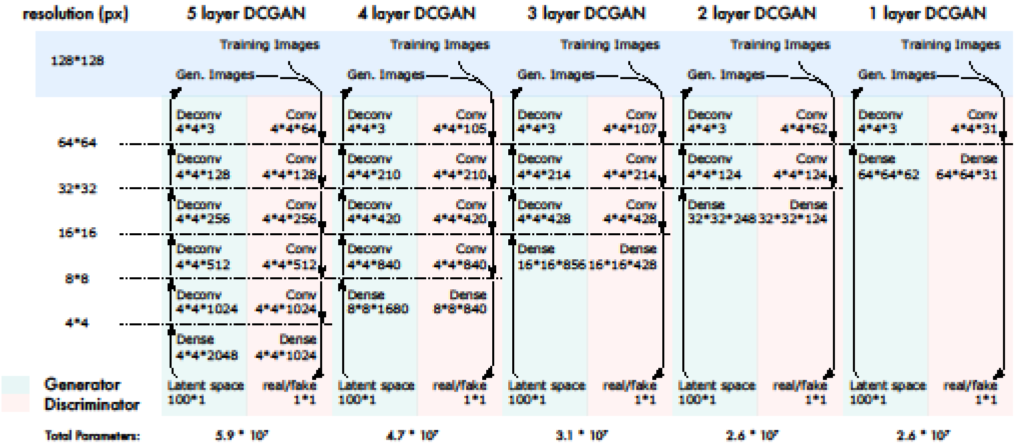

Figure S2:

DCGAN architectures. The parameters of convolutional and dense layers are shown for increasingly shallow DCGANs from left to right. Parameters were chosen so that image resolution would double between deconvolutional layers, processing depth (number of filters) would decrease by half for later deconvolutional layers and double for later convolutional layers. The latent space was always a vector of length 100.

Use of color information in our CNNs

To investigate the role of color in our CNNs, we took a set of 750 low- and 750 high-gloss images from the image set used for training DCGANs (not used for training the CNNs)

and passed these through the network both as RGB and as 3-channel grayscale inputs. Correlation between predictions for RGB and grayscale images per network were on average r = 0.90, 0.88, 0.91, 0.89, 0.89, 0.89, 0.89, 0.88, 0.87 for 1,2,3,4,5,6,7,8,12-layer networks respectively. Out of all 2639 networks we tested, only one had a correlation of less than 0.7 for colour and grayscale. See also **figure S3a**. This suggests a rather moderate role of colour relative to other cues. On average the networks have a lower accuracy with grayscale than with colour images. Network accuracy (the proportion of images judged correctly) dropped on average by 0.044, 0.059, 0.051, 0.064, 0.062, 0.062, 0.064, 0.067, 0.072 for 1,2,3,4,5,6,7,8,12-layer networks respectively. See **Figure S3b**. Taken together, these results indicate that grayscale images are judged slightly less accurately but for most networks this effect is rather small.

Supplementary Figure 3

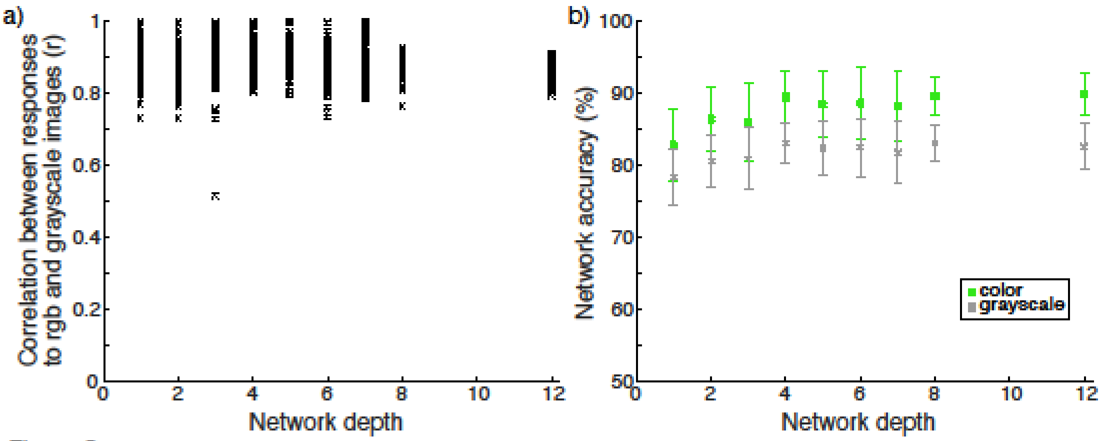

Figure S3:

a) Correlation between individual CNNs' responses to RGB and grayscale versions of the same 1 500 images (750 high gloss, 750 low gloss).
b) Mean network accuracy for the same RGB and grayscale images used in **figure S3a** for different network depths. Error bars show sd.

CNN accuracy outlier analysis

To address concerns that our analysis of CNN typicality (shown in **figure 2c and 2d**) is driven by outliers we performed two analyses.

In the first analysis we left out the least accurate 1,2,5,10 or 15 % of networks from each depth group to repeat the analysis without outliers. **Figure S4a** shows how the

distributions of networks of different depths in terms of accuracy and correlation to humans on the diagnostic image set change when the least accurate networks are left out of the analysis. **Figure S4b** shows the correlation coefficients of the individual depth groups in **figure S4a**, similar to**figure 2d**. We find similar results when we exclude the outliers. Deeper layers show a negative correlation between correlation to humans and accuracy. With increasing percentage removed from the dataset we find that shallower depth groups show a more negative correlation, moving the intersection with the x axis (the point of 0 correlation between accuracy and correlation to humans) towards shallower networks. Rejecting the least accurate 15% of networks results in an intersection with the x-axis between 1 and 2 layers.

In another analysis we took an alternative approach, looking within each depth group at the distance between the highest correlating network and the centroid of that depth group (**figure S4c**). Rather than a correlation as in the previous analysis, we look at the inverse gradient of these lines - the change of accuracy / the change in correlation to humans. The inverse gradients are shown against network depth in **figure S4d**. In this case, the x axis intersection of a fitted curve happens around 2 layers. At this point the estimated gradient is 0, meaning that networks of this depth can be more similar to humans independently from their accuracy.

Our concern with analyses leaving out or mitigating the effect of outliers is that the accuracy distributions appear to be systematically skewed - more so for shallow networks than for deep ones (see also **figure S4e**). Especially for 1-layer networks there appears to be a second cluster of less accurate networks. (dark blue, around 70% accuracy). Leaving out even the lowest 1% already affects this cluster. Omitting an increasing percentage of the least accurately performing networks affects depth groups differently and systematically. The deepest groups are hardly affected while the correlation of accuracy to correlation with humans becomes increasingly lower for shallower networks.

We therefore find that all networks with non-random responses should be part of the analysis, as reported in the main text. However, it is interesting to see that leaving out the least accurate networks still shows a similar trend in correlations between accuracy and correlations to humans. The main difference is that the point of 0 correlation shifts towards shallower networks.

Supplementary Figure 4

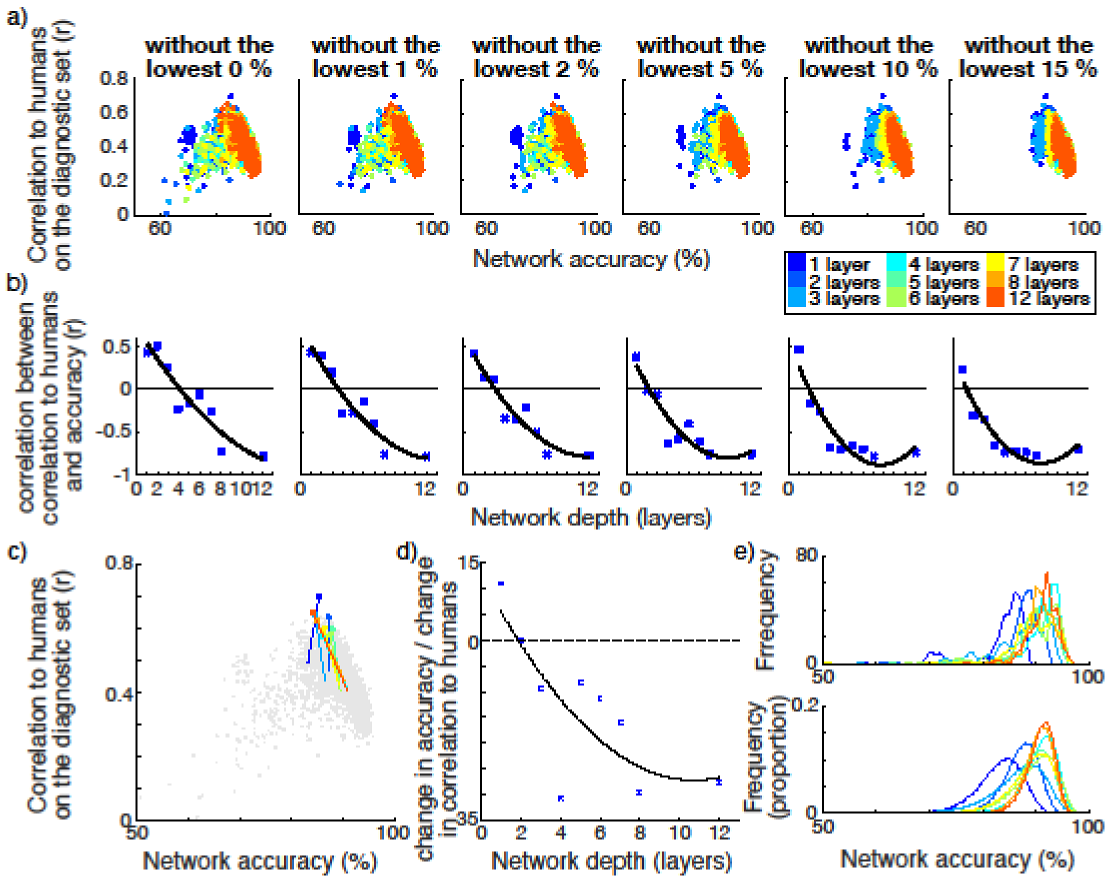

Figure S4:

a) CNNs from the Bayesian search in terms of accuracy and correlation to humans on the diagnostic image set, omitting an increasing percentage of the least accurate networks per depth group. The left-most plot shows the same data as **figure 2c** in the main text.
b) Correlation coefficients between accuracy and correlation to humans for each depth group, omitting ai increasing percentage of least accurate networks, corresponding to the plots in **figure S4a**
c) Lines from the centroid of each depth group to the network of that depth group that shows the highest correlation to humans (circles)
d) Inverse gradients of the lines shown in **figure S4c**. The pattern resembles that in **figure 2d** and **figure S4b**. The intersection of the fitted line with the x axis has a similar meaning – independence between network accuracy and correlation to humans – and is situated around 2 layers network depth.
e) Distributions of network accuracies. The top graph shows raw data, the bottom graph shows fitted distributions.
